# The impact of influenza on the health related quality of life in China: an EQ-5D survey

**DOI:** 10.1186/s12879-017-2801-2

**Published:** 2017-10-16

**Authors:** Juan Yang, Mark Jit, Yaming Zheng, Luzhao Feng, Xinxin Liu, Joseph T. Wu, Hongjie Yu

**Affiliations:** 1 School of Public Health, Fudan University, Key Laboratory of Public Health Safety, Ministry of Education, Shanghai, 200032 China; 2 Key Laboratory of Surveillance and Early-warning on Infectious Disease, Division of Infectious Disease, Chinese Center for Disease Control and Prevention, Beijing, 102206 China; 3 Modelling and Economics Unit, Public Health England, London, UK; 4 Department of Infectious Disease Epidemiology, London School of Hygiene and Tropical Medicine, London, UK; 5 Economics, Department of Economics, Emory University, Atlanta, USA; 6 WHO Collaborating Centre for Infectious Disease Epidemiology and Control, School of Public Health, Li Ka Shing Faculty of Medicine, The University of Hong Kong, Hong Kong Special Administrative Region, China

**Keywords:** Influenza, Health related quality of life, EQ-5D, China

## Abstract

**Background:**

Influenza causes considerable morbidity and mortality in China, but its impact on the health-related quality of life (HRQoL) has not been previously measured.

**Methods:**

We conducted a retrospective telephone survey to assess the impact of influenza on the HRQoL among outpatients and inpatients using the EuroQoL EQ-5D-3 L instrument. Participants were individuals with laboratory-confirmed influenza infection registered by the National Influenza-like-illness Surveillance Network in 2013.

**Results:**

We interviewed 839 of 11,098 eligible influenza patients. After excluding those who were unable to complete the HRQoL for the registered influenza episode, 778 patients were included in the analysis. Both outpatients (*n* = 529) and inpatients (*n* = 249) most commonly reported problems with pain/discomfort (71.8% of outpatients and 71.9% of inpatients) and anxiety/depression (62.0% of outpatients and 75.1% of inpatients). For individual influenza outpatients, the mean health utility was 0.6142 (SD 0.2006), and the average quality adjusted life days (QALD) loss was 1.62 (SD 1.84) days. The HRQoL of influenza inpatients was worse (mean health utility 0.5851, SD 0.2197; mean QALD loss 3.51 days, SD 4.25) than that of outpatients (*p* < 0.05). The presence of underlying medical conditions lowered the HRQoL for both outpatients and inpatients (p < 0.05).

**Conclusions:**

Influenza illness had a substantial impact on HRQoL. QALD loss due to an acute influenza episode in younger children was comparable to that due to enterovirus A71-associated hand, foot and mouth disease. Our findings are key inputs into disease burden estimates and cost-effectiveness evaluations of influenza-related interventions in China.

**Electronic supplementary material:**

The online version of this article (10.1186/s12879-017-2801-2) contains supplementary material, which is available to authorized users.

## Background

Influenza virus causes substantial morbidity and mortality in China, with an estimated 115–142 hospitalizations associated with severe acute respiratory infections (SARI) per 100,000 population, and 7.0–14.3 influenza associated respiratory and circulatory excess deaths per 100,000 persons every year [1, 2]. The World Health Organization recommends influenza vaccination for reducing influenza burden [3], but China has yet to implement a nationwide influenza vaccination programme for any risk group. To inform such a decision, the vaccine-preventable burden of influenza will need to be evaluated against other diseases with interventions that are competing for health care resources.

Previous reports have estimated influenza incidence and mortality, but none of them have investigated the impact of influenza on health-related quality of life (HRQoL) in China. HRQoL measures such as quality adjusted life years combine measures of morbidity and mortality into a single unit, hence enabling comparisons between diseases of varying incidence and severity. This information will be useful for parameterizing comparative analysis of interventions for influenza.

HRQoL can be measured using generic preference-based instruments such as the EuroQoL EQ-5D [4]. Two recent systematic reviews of studies measuring HRQoL associated with influenza [5, 6] had identified only seven original studies. The number of quality adjusted life days (QALDs) lost as a result of a single influenza episode (measured as 1/365 × quality adjusted life years lost) across these studies ranged widely between 1.68–17.34. None of the reviewed studies was conducted in China. HRQoL measurements from other countries is unlikely to be directly applicable in China because the utilities associated with different health states are likely to be influenced by cultural perceptions and preferences [7, 8].

To address this evidence gap, we conducted a survey to estimate the QALDs losses as a result of a single influenza episode resulting in either outpatient or inpatient hospital attendance. This provides essential information for decision makers to understand the magnitude of influenza burden in China compared to other diseases and evaluate interventions such as vaccination.

## Methods

A telephone investigation was conducted from 25 December 2013 to 11 January 2014 among influenza patients registered in the National Influenza-like-illness Surveillance Network (ILINet) in China. The survey was performed alongside a parallel survey among the same participants on the economic cost of influenza illness [9]. Participants were laboratory-confirmed influenza patients with landline or mobile phone numbers registered in the ILINet in 2013. We solicited the demographic (e.g. name, age, gender, address), clinical (e.g. date of onset, hospital visit date) and virological detection details of each patient from ILINet.

ILINet is a network of 408 provincial- and prefecture-level Centers for Disease Control and Prevention and 554 sentinel hospitals situated in 31 provinces [10]. It was established to monitor the activity, antigenic and genetic changes in seasonal influenza viruses in China. Specimens are then tested for influenza virus using cell culture and/or reverse transcription polymerase chain reaction (RT-PCR). Positive influenza specimens are typed and subtyped using RT-PCR. Assay kits and protocols follow guidelines released by the Chinese National Influenza Centre (a World Health Organization Collaborating Centre for Reference and Research on Influenza).

### EQ-5D instrument and telephone survey

The EuroQoL EQ-5D-3 L (hereafter just “EQ-5D”) is a widely used generic instrument for measuring non-disease-specific HRQoL. It comprises of a visual analogue scale (VAS) and a descriptive system. The VAS records the health status on a scale between zero (the worst imaginable health state) and 100 (the best imaginable health state). The EQ-5D descriptive system defines health in five dimensions: mobility, self-care, usual activities, pain/discomfort, and anxiety/depression. Each dimension has three categories: no problems, some problems and extreme problems. The health states defined by EQ-5D descriptive system can be converted into a numerical health utility score using country-specific value sets, by eliciting choices between different health states from each country’s populations, with zero representing death and one representing full health [4]. Negative values correspond to states considered to be less preferred than death.

We used the EQ-5D telephone interview version in simplified Chinese [11] for adult patients (aged 16 years and above) who could be reached by telephone. For patients younger than 16 years old and other patients for whom only the contact information of a caregiver was available, we used a slightly altered EQ-5D proxy version [11] (telephone-based proxy version: proxy 2, in which their caregivers were asked to rate how they believed the patients would rate their own HRQoL; available on request). For children younger than 18 months, the mobility and self-care dimensions were not scored because not all young children at these ages are able to walk and all of them are unable to care for themselves [12]. For these participants, the mobility and self-care dimensions were both given the value of “no problems” in the baseline analysis and “extreme problems” in a sensitivity analysis.

The telephone survey was conducted as follows: after obtaining verbal informed consent, a trained interviewer verified the respondent’s basic information recorded in the surveillance systems, including her/his name, age, gender, and the date of onset of illness. If verified and if the respondent was able to recall the influenza episode, the respondent was asked to complete the EQ-5D survey based upon the average day of their illness over the telephone, and to give the duration of influenza symptoms. Otherwise, she/he was excluded from the study. Each telephone number was called up to four times on different days before being classified as unreachable. A more detailed description of the study design has been reported in our earlier publication [9].

### Data analysis

Data analysis was performed using R version 3.0.3 [13]. Responses to the EQ-5D descriptive system were converted into health utilities using China-specific tariffs developed using regression models fitted to time trade-offs for respondents presented with different health states [14]. A range of models were fitted by the authors of *Chinese time trade-off values for EQ-5D health states* (Value Health 2014); we used the tariffs corresponding to the model using ordinary least squares regression on aggregate data, which had the lowest adjusted R^2^ of all the models (i.e., the “N3 model”) [14]. The model includes 11 dummy variables for specifying the problems in a health state: 1) with the MO2, SC2, UA2, PD2 and AD2 terms being 1(0) for the presence (absence) of moderate problems in mobility, self-care, usual activities, pain/discomfort, and anxiety/depression, respectively; 2) with MO3, SC3, UA3, PD3 and AD3 terms being 1(0) for the presence (absence) of extreme problems in these dimensions; 3) with N3 term being 1(0) for the presence (absence) of any extreme problems; 4) coefficients for MO2, SC2, UA2, PD2, AD2, MO3, SC3, UA3, PD3, AD3 and N3 were 0.099, 0.105, 0.074, 0.092, 0.086, 0.246, 0.208, 0.193, 0.236, 0.205, 0.022, respectively; 5) with a constant coefficient being 0.039 [14]. Utility scores were then converted into QALD losses by multiplying the difference in health utility during the influenza episode from background health utility by the reported duration of illness. The health utility of general population across China was not available. We used the average (0.86) of background health utility of rural residents in western China [15] and influenza patients from other countries (UK and Spain) [5, 15–17] as controls to compare with that of influenza patients in our study (more details shown in Additional file 1).

Results were stratified by age, gender, risk status (high risk patients refer to those with underlying medical conditions including: chronic respiratory disease, asthma, chronic cardiovascular diseases, diabetes, chronic liver disease, and chronic renal disease, etc. Other patients without these underlying diseases are low risk patients [3]), urban or rural residence (as previously defined [9]), economic development region (East, Central, and West) (see Additional file 2: Fig. S1) [18], hospital level (distinguished by numbers of beds, health-care workers, facilities and equipment, etc., as previously defined [9] and details also shown in Additional file 3), and virus type (influenza A, influenza B, and influenza untyped, with the latter corresponding to samples for which influenza virus typing was not conducted).

We used bootstrap multiple linear regression (with 1000 replications) to analyse the determinants of the VAS score, as well as health utility and QALD loss calculated from the descriptive system, in other words, to evaluate the role of socio-demographic (e.g., age and gender) and other variables (e.g., risk status, influenza type) on the rating of VAS score, health utility and on QALD loss. The Chi-Square test or Fisher’s Exact test were used for qualitative variables. It was found that population structure (by age group and levels of hospitals) of included patients was significantly different from that of other influenza patients from the National ILINet (details shown in Additional file 4: Table S1). Hence we calculated the weighted HRQoL of included patients using the population structure of the surveillance network as a reference.

## Results

### Characteristics of enrolled patients

Of 39,968 laboratory-confirmed influenza patients registered in the National ILINet, 11,098 influenza patients with telephone numbers registered were eligible, and 778 patients who were successfully interviewed (529 outpatients and 249 inpatients) were included in the analysis. Statistically significant differences in age and hospital level but not in gender and region were observed between included (*n* = 778) and excluded influenza patients (*n* = 39,190) from the surveillance network (Additional file 4: Table S1). Further details including a flow diagram of participant selection have been previously published [9].

The median age of included respondents was seven years (interquartile range, IQR 3–15), with 589 (75.7%) respondents younger than 16 years of age. 438 (82.8%) outpatients and 166 (66.7%) inpatients were from urban areas. 117 (22.1%) outpatients and 131 (52.6%) inpatients had underlying medical conditions. 271 (51.2%) outpatients and 113 (45.4%) inpatients were from East China. (Table 1) The average duration of an influenza episode was 6.2 (standard deviation, SD 3.5) days and 11.8 (SD 7.5) days respectively for influenza outpatients and inpatients.

**Table 1:** Characteristics of included influenza patients in the health-related quality of life survey, China, 2013 (n, %)

Characteristics	Influenza outpatients (*n* = 529)	Influenza inpatients (*n* = 249)	Total (*n* = 778)
Median age, years (IQR) ^a^	8 (5–20)	4 (2–7)	7 (3–15)
Age group, years			
< 5	122 (23.1%)	141 (56.6%)	263 (33.8%)
5–15	246 (46.5%)	80 (32.1%)	326 (41.9%)
16–59	146 (27.6%)	24 (9.6%)	170 (21.9%)
≥ 60	15 (2.8%)	4 (1.6%)	19 (2.4%)
Male	281 (53.1%)	144 (57.8%)	425 (54.6%)
Risk status ^b^			
Low-risk	412 (77.9%)	118 (47.4%)	530 (68.1%)
High-risk	117 (22.1%)	131 (52.6%)	248 (31.9%)
Area			
Urban area	438 (82.8%)	166 (66.7%)	604 (77.6%)
Rural area	91 (17.2%)	83 (33.3%)	174 (22.4%)
Region			
East China	271 (51.2%)	113 (45.4%)	384 (49.4%)
Central China	122 (23.1%)	78 (31.3%)	200 (25.7%)
West China	136 (25.7%)	58 (23.3%)	194 (24.9%)
Hospital ^c^			
Level 3	298 (56.3%)	177 (69.7%)	475 (60.7%)
Level 2	119 (22.5%)	58 (22.8%)	177 (22.6%)
Level 1 and lower	112 (21.2%)	19 (7.5%)	131 (16.7%)
Virus type			
Untyped ^d^	307 (58.0%)	183 (73.5%)	490 (63.0%)
Influenza A	164 (31.0%)	30 (12.0%)	194 (24.9%)
Influenza B	58 (11.0%)	36 (14.5%)	94 (12.1%)

^a^ IQR: inter-quartile range

^b^ Risk status: high risk patients refer to those with underlying medical conditions including: chronic respiratory disease, asthma, chronic cardiovascular diseases, diabetes, chronic liver disease, and chronic renal disease, etc. Other patients without these underlying diseases are low risk patients

^c^ Level 3 is the top level, followed by level 2 and level 1 in order

^d^ Untyped: Laboratory tests for influenza virus type identification were not conducted

### EQ-5D dimension results

**Fig. 1:**
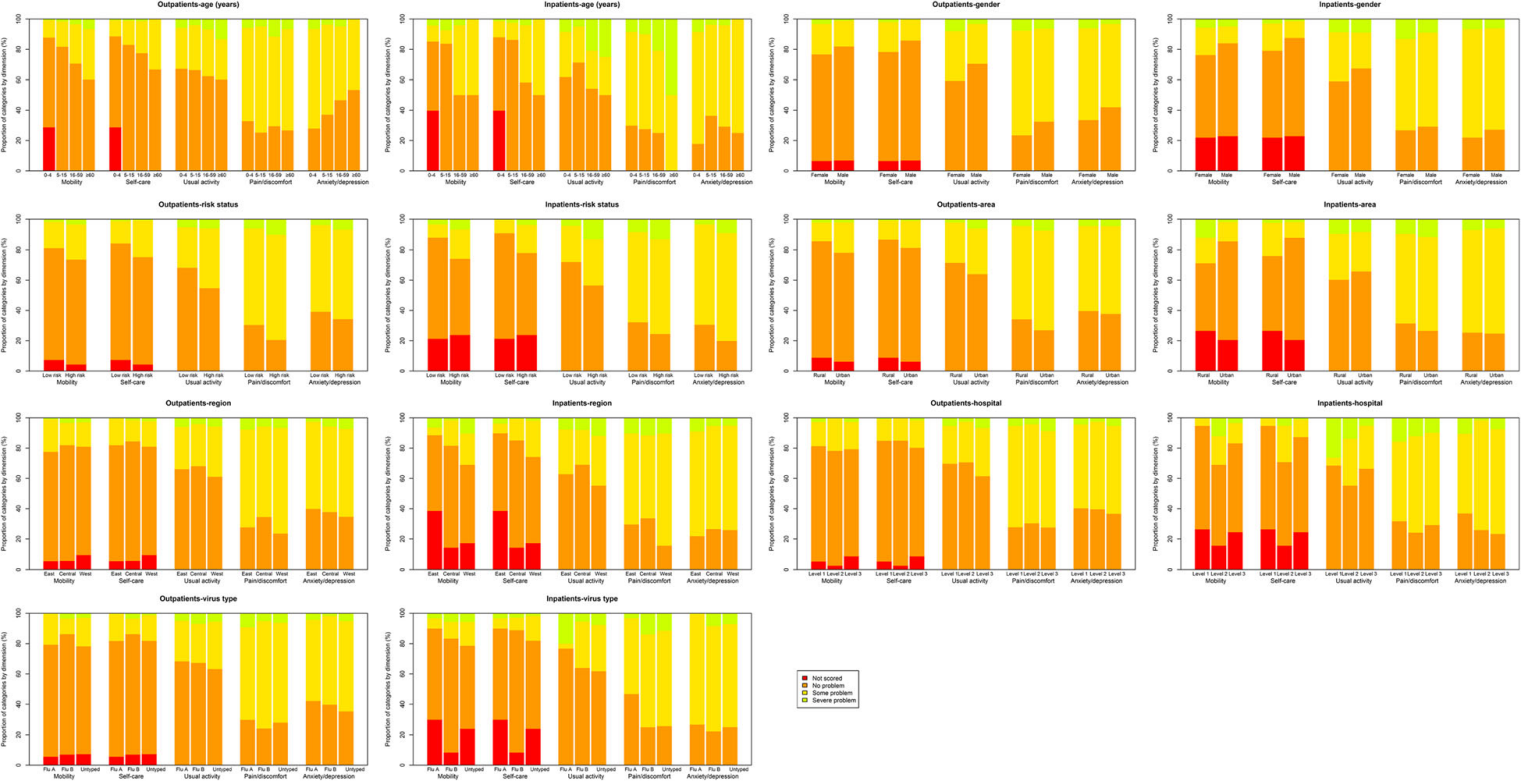
Ratings by EQ-5D dimension for influenza outpatients and inpatients. Not scored: For all the influenza patients younger than 18 months, the mobility and self-care dimensions were not scored and assumed to be no problem in the baseline analysis of health-related quality of life

Both influenza outpatients and inpatients reported problems on all five dimensions measured in the EQ-5D. Problems with pain/discomfort (71.8% of outpatients and 71.9% of inpatients) and anxiety/depression (62.0% of outpatients and 75.1% of inpatients) were the most commonly reported. Relatively fewer patients (below 40% of both outpatients and inpatients) reported problems with mobility, self-care, and usual activity. The proportions of patients reporting severe problems on any dimension were quite low (below 10%, apart from pain/discomfort). Problems with pain/discomfort and anxiety/depression were also the most commonly reported across different strata of patients. (Fig. 1).

### HRQoL of influenza patients

Average values for VAS, health utilities and QALD losses for both inpatients and outpatients are shown in Table 2. The HRQoL of inpatients was significantly worse on all three measures (*p* < 0.05). When weighted by population structure (in terms of age and hospital level), the reported HRQoL of outpatients and inpatients changed slightly. Multiple linear regression indicated having underlying diseases was significantly associated with greater QALD losses in both outpatients and inpatients (p < 0.05). In addition, being female or from West China was significantly associated with greater QALD losses in outpatients, and less QALD losses observed in influenza A inpatients. More details were presented in Tables 3 and 4.

**Table 2:** Health-related quality of life of influenza outpatients and inpatients

	n	VAS		Health utility		QALD loss ^a^	
		Mean (SD)	Median (IQR)	Mean (SD)	Median (IQR)	Mean (SD)	Median (IQR)
Influenza outpatient							
Unweighted	529	68.93 (19.08)	70.00 (60.00, 80.00)	0.6142 (0.2006)	0.6430 (0.5630, 0.7350)	1.62 (1.84)	1.08 (0.46, 2.04)
Weighted^b^	529	68.20 (35.98)	66.18 (42.05, 87.34)	0.6087 (0.3518)	0.5678 (0.3333, 0.7532)	1.69 (2.52)	0.94 (0.39, 1.94)
Influenza inpatient							
Unweighted	249	65.94 (20.61)	70.00 (50.00, 80.00)	0.5851 (0.2197)	0.6430 (0.4770, 0.7290)	3.51 (4.25)	2.17 (1.00, 4.45)
Weighted^b^	249	64.07 (29.03)	60.83 (43.45, 78.22)	0.5616 (0.2799)	0.5588 (0.3846, 0.6925)	3.63 (5.53)	2.03 (0.80, 3.93)

^a^ QALD: quality-adjusted life days

^b^ Weighted: Previous analysis showed that population structure (by age group and levels of hospitals) of included patients was significantly different from that of other influenza patients from the National ILI Surveillance Network [Additional file 4: Table S1]. Hence we calculated the weighted HRQoL of included patients using the population structure of the National ILI Surveillance Network as a reference

**Table 3:** Health-related quality of life of influenza outpatients

	n	VAS			Health utility			QALD loss ^a^		
		Mean (SD)	Median (IQR)	Multiple linear regression (95% CI) ^b^	Mean (SD)	Median (IQR)	Multiple linear regression (95% CI) ^b^	Mean (SD)	Median (IQR)	Multiple linear regression (95% CI) ^b^
Total	529	68.93 (19.08)	70.00 (60.00, 80.00)		0.6142 (0.2006)	0.6430 (0.5630,0.7350)		1.62 (1.84)	1.09 (0.46,2.04)	
Age group (years)										
0–4	122	71.39 (20.06)	75.00 (60.00, 80.00)	Reference	0.6286 (0.1963)	0.6430 (0.5690,0.7350)	Reference	1.72 (1.84)	1.48 (0.65,2.15)	Reference
5–15	246	69.65 (18.40)	70.00 (60.00, 80.00)	−2.56 (−6.79, 1.80)	0.6216 (0.1921)	0.6430 (0.5690,0.7290)	−0.0119 (−0.0525, 0.0332)	1.47 (1.53)	1.09 (0.50,1.98)	−0.13 (−0.52, 0.20)
16–59	146	65.53 (19.71)	70.00 (50.00, 80.00)	−5.88 (−10.73, −0.94)^f^	0.5939 (0.2165)	0.6430 (0.4575,0.7290)	−0.0329 (−0.0840, 0.0175)	1.77 (2.31)	0.92 (0.40,2.33)	0.13 (−0.33, 0.66)
≥ 60	15	70.33 (10.77)	70.00 (65.00, 72.50)	0.22 (−6.55, 7.65)	0.5733 (0.2109)	0.6430 (0.4130,0.7290)	−0.0424 (−0.1525, 0.0760)	1.84 (1.47)	1.27 (0.92,3.00)	0.01 (−0.79, 0.84)
Gender						*p* = 0.0041			*p* = 0.0441	
Female	248	67.05 (20.51)	70.00 (50.00, 80.00)	Reference	0.584 (0.2164)	0.6430 (0.4510,0.7290)	Reference	1.79 (1.95)	1.30 (0.52,2.17)	Reference
Male	281	70.59 (17.58)	70.00 (60.00, 80.00)	3.26 (−0.01, 6.39)	0.6408 (0.1818)	0.6430 (0.5690,0.7350)	0.0565 (0.0216, 0.0900)^f^	1.47 (1.73)	1.09 (0.39,1.98)	−0.32 (−0.62, −0.02)^f^
Risk status ^c^						*p* = 0.0097			*p* < 0.001	
Low-risk	412	69.88 (18.87)	70.00 (60.00, 80.00)	Reference	0.6267 (0.1948)	0.6430 (0.5690,0.7350)	Reference	1.42 (1.63)	0.95 (0.39,1.98)	Reference
High-risk	117	65.58 (19.50)	70.00 (50.00, 80.00)	−4.36 (−8.20, −0.55)^f^	0.5698 (0.2147)	0.6430 (0.3650,0.7290)	−0.0573 (−0.1029, −0.0158)^f^	2.33 (2.32)	1.74 (0.88,2.87)	0.87 (0.51, 1.39)^f^
Area										
Urban	438	68.28 (19.49)	70.00 (60.00, 80.00)	Reference	0.6075 (0.2041)	0.6430 (0.5432,0.7290)	Reference	1.67 (1.89)	1.16 (0.50,2.07)	Reference
Rural	91	72.09 (16.68)	75.00 (60.00, 80.00)	2.71 (−1.84, 6.50)	0.646 (0.1802)	0.6430 (0.5690,0.7350)	0.0339 (−0.0080, 0.0728)	1.40 (1.56)	0.92 (0.39,1.97)	−0.26 (−0.63, 0.13)
Region									*p* = 0.0373	
East China	271	68.79 (19.43)	70.00 (60.00, 80.00)	Reference	0.6162 (0.1905)	0.6430 (0.5440,0.7290)	Reference	1.53 (1.80)	1.09 (0.43,1.99)	Reference
Central China	122	69.48 (19.56)	70.00 (60.00, 80.00)	0.08 (−4.43, 4.21)	0.6298 (0.217)	0.6430 (0.5690,0.7470)	0.0064 (−0.0404, 0.0512)	1.50 (1.80)	1.09 (0.16,2.03)	0.01 (−0.32, 0.48)
West China	136	68.74 (18.04)	70.00 (60.00, 80.00)	−1.26 (−4.87, 2.77)	0.596 (0.2051)	0.6430 (0.5372,0.7290)	−0.0287 (−0.0705, 0.0082)	1.92 (1.94)	1.37 (0.65,2.52)	0.45 (0.11, 0.89)^f^
Hospital ^d^										
Level 3	298	67.23 (19.67)	70.00 (60.00, 80.00)	Reference	0.6006 (0.2171)	0.6430 (0.4922,0.7335)	Reference	1.82 (2.13)	1.30 (0.46,2.17)	Reference
Level 2	119	71.18 (17.39)	70.00 (60.00, 80.00)	3.61 (−0.53, 7.19)	0.6364 (0.1575)	0.6430 (0.5690,0.7350)	0.0259 (−0.0160, 0.0625)	1.23 (1.10)	0.92 (0.48,1.6)	−0.46 (−0.80, −0.15)^f^
Level 1 and lower	112	71.06 (18.89)	72.50 (60.00, 85.00)	2.96 (−1.50, 7.42)	0.6266 (0.1944)	0.6430 (0.5690,0.7350)	0.0188 (−0.0250, 0.0607)	1.51 (1.56)	1.11 (0.49,2.07)	−0.25 (−0.63, 0.08)
Virus type										
Untyped ^e^	307	67.59 (19.59)	70.00 (60.00, 80.00)	Reference	0.6072 (0.2052)	0.6430 (0.5440,0.7350)	Reference	1.67 (1.92)	1.09 (0.50,2.04)	Reference
Influenza A	164	70.21 (17.98)	70.00 (60.00, 80.00)	3.22 (−0.60, 6.54)	0.6242 (0.1889)	0.6430 (0.5430,0.7350)	0.0217 (−0.0147, 0.0580)	1.56 (1.79)	1.11 (0.39,2.17)	−0.11 (−0.45, 0.24)
Influenza B	58	72.41 (18.95)	70.00 (60.00, 85.00)	4.31 (−1.54, 9.36)	0.6227 (0.21)	0.7290 (0.5690,0.7290)	0.0137 (−0.0489, 0.0639)	1.54 (1.54)	0.92 (0.52,1.95)	−0.12 (−0.55, 0.32)

^a^ QALD: quality-adjusted life days

^b^ Compared to the reference, absolute increase or decrease of the VAS, health utility and/or QALD loss. And we obtained the bias-corrected and accelerated (BCa) bootstrap percentile confidence interval using the R function “boot.ci”

^c^ Risk status: high risk patients refer to those with underlying medical conditions including: chronic respiratory disease, asthma, chronic cardiovascular diseases, diabetes, chronic liver disease, and chronic renal disease, etc. Other patients without these underlying diseases are low risk patients

^d^ Level 3 is the top level, followed by level 2 and level 1 in order

^e^ Untyped: Laboratory tests for influenza virus type identification were not conducted

^f^ p < 0.05: significant differences

**Table 4:** Health-related quality of life of influenza inpatients

	N	VAS			Health utility			QALD loss^a^		
		Mean (SD)	Median (IQR)	Multiple linear regression (95%CI) ^b^	Mean (SD)	Median (IQR)	Multiple linear regression (95%CI) ^b^	Mean (SD)	Median (IQR)	Multiple linear regression (95%CI) ^b^
Total	249		65.94 (20.61)	70.00 (50.00, 80.00)	0.5851 (0.2197)	0.6430 (0.4770,0.7290)		3.51 (4.25)	2.17 (1.00,4.45)	
Age group (years)									*p* = 0.0431	
0–4	141	65.76 (20.68)	70.00 (50.00,80.00)	Reference	0.5900 (0.2025)	0.6430 (0.5440,0.7350)	Reference	3.51 (3.96)	2.39 (1.12,4.75)	Reference
5–15	80	67.00 (19.87)	70.00 (50.00,80.00)	0.28 (−6.44,5.91)	0.6132 (0.2185)	0.6430 (0.5690,0.7305)	0.0386 (−0.0205,0.0973)	2.75 (3.27)	1.95 (0.62,3.45)	−0.91 (−1.93,0.08)
16–59	24	63.67 (23.93)	60.00 (50.00,90.00)	−2.07 (−11.72,7.10)	0.4913 (0.2828)	0.5965 (0.2240,0.6645)	−0.0847 (−0.1904,0.0114)	5.16 (6.07)	2.59 (1.46,7.11)	1.49 (−0.69,4.16)
≥60^c^	4	65.00 (17.32)	65.00 (50.00,80.00)	−2.07 (−11.72,7.10)	0.4128 (0.2690)	0.4210 (0.2938,0.5400)	−0.0847 (−0.1904,0.0114)	8.98 (10.79)	4.83 (3.49,10.32)	1.49 (−0.69,4.16)
Gender										
Female	105	63.27 (21.24)	70.00 (50.00,80.00)	Reference	0.5633 (0.2380)	0.6430 (0.4280,0.7290)	Reference	3.64 (4.07)	2.60 (1.05,4.45)	Reference
Male	144	67.9 (19.99)	70.00 (50.00,80.00)	5.11 (−0.64,10.66)	0.6010 (0.2046)	0.6430 (0.5380,0.7350)	0.0471 (−0.0110,0.1017)	3.41 (4.38)	2.04 (0.91,4.39)	−0.46 (−1.38,0.58)
Risk status ^d^			*p* = 0.0347			*p* = 0.0022			*p* < 0.001	
Low-risk	118	68.91 (20.75)	70.00 (50.00,80.00)	Reference	0.6340 (0.1774)	0.6430 (0.5690,0.7350)	Reference	2.18 (2.26)	1.63 (0.87,3.04)	Reference
High-risk	131	63.27 (20.2)	60.00 (50.00,80.00)	−5.8 (−10.90, −1.00)g	0.5411 (0.2442)	0.6300 (0.4155,0.6490)	−0.0943 (−0.1455, −0.0412)^g^	4.71 (5.17)	3.25 (1.52,6.46)	2.47 (1.61, 3.62)^g^
Area										
Urban	166	64.27 (20.89)	70.00 (50.00,80.00)	Reference	0.5993 (0.2018)	0.6430 (0.5488,0.7290)	Reference	3.20 (3.40)	2.07 (1.25,4.37)	Reference
Rural	83	69.3 (19.75)	70.00 (50.00,80.00)	5.11 (−0.34,10.57)	0.5566 (0.2505)	0.6430 (0.3840,0.7350)	−0.0390 (−0.1051,0.0197)	4.14 (5.54)	2.60 (0.87,4.86)	0.93 (−0.16,2.24)
Region										
East China	113	66.58 (21.65)	70.00 (50.00,80.00)	Reference	0.6066 (0.2101)	0.6430 (0.5200,0.7350)	Reference	3.36 (4.35)	1.98 (0.88,4.21)	Reference
Central China	78	64.5 (19.46)	65.00 (50.00,80.00)	−2.67 (−9.21,3.46)	0.5917 (0.2194)	0.6430 (0.5690,0.7290)	−0.0211 (−0.0895,0.0422)	3.02 (2.89)	2.25 (1.01,4.35)	−0.27 (−1.51,0.81)
West China	58	66.64 (20.29)	70.00 (50.00,80.00)	−1.27 (−8.85,5.80)	0.5343 (0.2337)	0.5815 (0.3808,0.6430)	−0.0774 (−0.1488, −0.0033)^g^	4.47 (5.35)	2.64 (1.36,5.44)	1.21 (−0.34,2.87)
Hospital ^e^										
Level 3	172	65.62 (19.88)	70.00 (50.00,80.00)	Reference	0.6003 (0.1956)	0.6430 (0.5440,0.7350)	Reference	3.19 (3.53)	2.12 (1.04,4.02)	Reference
Level 2	58	66.43 (22.89)	70.00 (50.00,80.00)	0.69 (−6.22,7.34)	0.5339 (0.2797)	0.6430 (0.3650,0.7290)	−0.0574 (−0.1382,0.0129)	4.59 (5.96)	2.57 (1.25,5.5)	1.33 (−0.10,3.44)
Level 1 and lower	19	67.37 (20.84)	70.00 (50.00,80.00)	0.80 (−11.79,11.42)	0.6032 (0.2065)	0.6430 (0.4955,0.7380)	0.0100 (−0.0941,0.0998)	3.08 (3.58)	1.95 (0.83,4.6)	−0.29 (−1.95,1.76)
Virus type										
Untyped ^f^	183	64.96 (19.94)	70.00 (50.00,80.00)	Reference	0.5770 (0.2220)	0.6430 (0.4770,0.7290)	Reference	3.74 (4.63)	2.17 (1.07,4.86)	Reference
Influenza A	30	67.83 (22.12)	70.00 (52.50,80.00)	0.63 (−8.18,10.13)	0.6382 (0.2139)	0.6430 (0.5875,0.7350)	0.0575 (−0.0335,0.1317)	2.35 (2.52)	1.52 (0.50,3.25)	−1.45 (−2.94, −0.14)^g^
Influenza B	36	69.39 (22.73)	77.50 (56.00,86.25)	3.55 (−4.96,11.79)	0.5821 (0.2119)	0.6430 (0.4648,0.7290)	−0.0301 (−0.1191,0.0390)	3.29 (3.07)	2.71 (1.11,3.98)	0.25 (−0.91,1.42)

^a^ QALD: quality-adjusted life days

^b^ Compared to the reference, absolute increase or decrease of the VAS, health utility and/or QALD loss. And we obtained the bias-corrected and accelerated (BCa) bootstrap percentile confidence interval using the R function “boot.ci”

^c^ 4 patients in the ≥60 age group were grouped into 15–59 years of age group in the multivariable linear regression analysis

^d^ Risk status: high risk patients refer to those with underlying medical conditions including: chronic respiratory disease, asthma, chronic cardiovascular diseases, diabetes, chronic liver disease, and chronic renal disease, etc. Other patients without these underlying diseases are low risk patients

^e^ Level 3 is the top level, followed by level 2 and level 1 in order

^f^ Untyped: Laboratory tests for influenza virus type identification were not conducted

^g^ p < 0.05: significant differences

There were 35 (6.6%) influenza outpatients and 56 (22%) influenza inpatients younger than 18 months. The mobility and self-care domains of these patients were not elicited, and were given scores corresponding to no problems and severe problems respectively in the baseline analysis and sensitivity analysis. HRQoL measures were much more severe in the sensitivity analysis in both outpatients (health utility 0.1651, QALD loss 5.05 compared to health utility 0.6235, QALD loss 1.78) and inpatients (health utility 0.1556, QALD loss 8.72 compared to health utility 0.6152, QALD loss 3.11). However, due to the small proportions of these younger patients, we found that the uncertainty in their mobility and self-care domains had slightly impact on HRQoL for all outpatients (health utility 0.5838, QALD loss 1.84 compared to health utility 0.6142, QALD loss 1.62) and inpatients (health utility 0.4817, QALD loss 4.77 compared to health utility 0.5851, QALD loss 3.51).

In patients aged 16 years and above, there were no significant differences in VAS, health utility or QALD loss between those who filled in the EQ-5D for themselves and those who had it filled in by proxy (*p* > 0.05). (Additional file 5: Table S2).

## Discussion and conclusion

This is the first study to explore the HRQoL impact of influenza in China. We found influenza was associated with a mean QALD loss of 1.62 in outpatients and 3.51 in inpatients. In the children younger than five years, the QALD loss during an influenza episode was 1.72 days for outpatients and 3.51 days for inpatients (see Tables 3 and 4). This is similar to the HRQoL impact of other acute infectious diseases of national importance in children such as enterovirus 71-associated hand, foot and mouth disease (with QALD loss of 1.31–2.99 days for outpatients, and 5.44 days for inpatients [19]). Although the duration of influenza symptoms is much shorter, the health utility of patients during an acute influenza episode (≥60 years old: 0.4128–0.5733, and 16–59 years old: 0.4913–0.5939) is worse than that of patients with chronic diseases such as diabetes (≥60 years old: 0.865, and 18–59 years old: 0.902 [20]) and active chronic hepatitis B (≥18 years old: 0.773 [21]). Some of the influenza patients included in our analysis may have complications. It was expected that HRQoL of complicated influenza patients was worse than that of uncomplicated influenza. However, we were unable to distinguish HRQoL between influenza patients with and without complications since the complications were not available for the patients from ILINet. The HRQoL stratified by complicated and uncomplicated influenza patients merit further investigation in the future.

We used the standard EQ-5D instrument (telephone version) for 76.0% of adult patients aged 16 years and above who could be reached by telephone, and used the EQ-5D telephone-based proxy version for 24.0% of adult patients for whom only the caregivers’ contact information is available and for all children younger than 16 years of age. We compared the HRQoL of adult patients between the self-reported and proxy-reported groups, and found that the EQ-5D telephone-based proxy version gave similar values of HRQoL when compared to the standard EQ-5D telephone version for adult patients (when factors like age, gender, presence of underlying diseases were controlled for). Previous publications found that proxy EQ-5D responses only had a mild to moderate agreement with the patients’ responses [22–26]. Compared to them, our study seems to have performed better in this respect than other proxy studies. We note that the EQ-5D is generally thought to be valid only for populations older than 12 years and the EQ-5D-Y only for children older than 8 years [27]. For other younger children, the reliability and validity of child-report health information are often questinable due to their developmental stage, language ability, and congitive functioning [28]. Therefore, we used the EQ-5D proxy version as an alternative in this study to rate the quality of life for all children.

A study on the effect of measles on HRQoL in UK found that 40% and 70% of proxy respondents did not answer the mobility and self-care dimensions respectively in the EQ-5D questionnaire to evaluate a young child’s HRQoL [12]. Therefore, the mobility and self-care dimensions for influenza patients younger than 18 months were not scored in our telephone survey. Sensitivity analysis showed that the HRQoL for these outpatients younger than 18 months varied greatly when these younger children were assumed to be severe problem compared to no problem. However, the overall HRQoL of all influenza outpatients and inpatients was not sensitive to variations in this assumption due to the small proportions of patients younger than 18 months in the surveillance network datasets.

Compared to influenza patients from the National ILINet who did not participate in the study, more influenza patients included in our analysis were younger than 16 years old (75.7% vs. 61.1%). Due to unavailability of characteristics of all influenza patients at a national level, we could not precisely determine how well our sample represents all influenza patients in China and not generalize our results to that at the national level. Instead, we weighted HRQoL of included patients using the population structure of the National ILINet as a reference, in order that our results could represent the HRQoL of all influenza patients registered in the National ILINet. Moreover, the multiple linear regression showed that no significant differences in HRQoL was observed between age groups (Tables 3 and 4). Thus, even if the children were over-represented, it would not have a big impact on the HRQoL of overall study participants. Another point of concern was that the high-level hospitals were overrepresented in the surveillance system, which do not cover basic medical institutions [9]. Patients with severe influenza are more likely to seek medical care in high-level hospitals than those with mild influenza who prefer basic medical institutions or practice self-care at home. Accordingly, patients with severe influenza may be overrepresented in our survey, which may result in an overestimation of the impact of influenza on HRQoL.

This study has several limitations. First, we asked respondents to evaluate their average HRQoL of all days during their influenza episode (other than that for a specific day like the worst day for the illness) and assumed that the health utility dropped to this level from day one of infection and remained constant, then returned to its highest level upon recovery. It was likely the study participants rated the impact of influenza infection on HRQoL for the worst day of infection instead. In this case, we assumed that the impact of influenza infection on HRQoL can be described by a triangular shape, which means the health utility linearly dropped to the worst level, and then linearly increased to the highest level. This reduced the QALD loss by half. Ideally, we would collect HRQoL information prospectively from patients, which would allow us to take several measurements to track how HRQoL changes over the course of the influenza episode, allowing more accurate estimation of QALD loss. Further prospective studies in the direction should make sense.

Second, it was impossible for us to rate the background health utility before influenza infections in the retrospective study, and there were no datasets measuring the HRQoL of the general population across China that could be used as controls to compare with our sample of patients with influenza. Hence, we calculated the QALD losses of influenza patients based on the difference between their health utility and the average of other populations (health utility: 0.86) [5, 15–17]. Due to the potential difference of health status and cultural perceptions between our study population and rural residents in western China as well as population from other countries, the estimation of QALD losses would be biased. As aforementioned, future prospective studies with several measurements during and after an influenza episode is necessary.

Third, since National ILINet report patients with elevated temperature. However, a substantial population of influenza patients aged 60 years old and above do not have fever [29, 30]. That may be related to the very low proportion (4.1%) of elderly influenza patients reported in the surveillance network [9], and accordingly result in limited number (*n* = 19, 2.4% of all study population) of elderly participants in our study. Because the elderly were more likely to have underlying medical conditions which have been found to be significantly related with greater QALD losses in this study, we may underestimate the impact of influenza on HRQoL with limited number of elderly participants.

Fourth, our respondents may have been subject to recall bias since they were asked to complete the EQ-5D retrospectively (The time between the influenza episode and interview was less than 46 days for 50% of our study participants [9]) rather than during their episode. We split study population into four groups on the basis of the 25th, 50th, 75th and 100th percentiles of the time delay between survey and the actual influenza episode, and did not found significant difference in HRQoL between them (data shown in Additional file 6: Table S3). Lastly, a high proportion (32%) of participants were influenza inpatients in our study. It was found that the HRQoL of influenza inpatients was much more impacted in comparison with influenza outpatients, with worse health utility and higher QALD loss. It revealed that more severe influenza patients may be more willing to take part in the survey. That maybe probably because they have a deeper insight and memory into the episode of influenza infection. The possible selection bias may lead to overestimating the health impact of influenza infections.

Despite these limitations, this study is the first attempt to measure the HRQoL of influenza patients in China. Influenza illness had a substantial impact on HRQoL. Our findings are important inputs for burden of disease studies and health economic evaluations of influenza-related interventions such as vaccination. These in turn are essential to inform decision making on the distribution of health care resources, since they enable comparisons between diseases of varying incidence and severity.

## Additional files

Additional file 1: Overview of published estimates of background health weight for influenza patients. (DOCX 23 kb)

Additional file 2: Figure S1. Map of geographic regions in mainland China. (TIFF 1033 kb)

Additional file 3: Definition of hospital levels. (DOCX 19 kb)

Additional file 4: Table S1. Characteristics of included patients and other lab-confirmed influenza patients from the National ILI Surveillance Network in the survey. (DOCX 25 kb)

Additional file 5: Table S2. Comparison of the health-related quality of life for influenza patients aged 16 years and above between the self-report and proxy-report groups. (DOCX 22 kb)

Additional file 6: Table S3. Health-related quality of life of influenza patients stratified by time delay between survey and the influenza episode. (DOCX 32 kb)

## Abbreviations

EQ-5D: EuroQoL EQ-5D-3 L
HRQoL: Health-related quality of life
ILINet: National Influenza-like-illness Surveillance Network
QALDs: Quality adjusted life days
RT-PCR: Reverse transcription polymerase chain reaction
SARI: Severe acute respiratory infections
VAS: Visual analogue scale
