# Supplementary material for: The impact of influenza on the health related quality of life in China: an EQ-5D survey
Source: BMC Infect Dis. 2017 Oct 16;17:686. doi: 10.1186/s12879-017-2801-2 (PMC5644056; doi:10.1186/s12879-017-2801-2)
Supplement: Supplementary file 1 — Overview of published estimates of background health weight for influenza patients. (DOCX 23 kb) [file 12879_2017_2801_MOESM1_ESM.docx]

**Additional file 1.** **Overview of published estimates of background health weight for influenza patients**

Van Hoek AJ, et al, performed a literature review on the estimates of the quality of life detriment due to influenza in PubMed, with search terms “influenza” and “quality-adjusted life year’, “QALY”, “QALD” or “EQ-5D”. It showed that only one original studies reported the background health weight till 2010. [1] We searched literatures from 2011 till August 2017 in PubMed using the same search terms, and found two more studies. Additionally, we searched published health utility of general population in China in CNKI and Wanfang. Summary of the studies was shown below. Averagely, background health utility for influenza patients was 0.86.

| Study | Country | Study participants | Background QALY weight |
| --- | --- | --- | --- |
| Griffin, AD  (2001) [2] | UK | - Confirmed influenza patients (within 3 months after onset of ILI, aged 18 years old and above): n=21 - General practitioner assessment of hypothetical high-risk influenza patient: n=8 | - Confirmed influenza patients: 0.817 - General practitioner assessment: 0.72 |
| Van Hoek, AJ  (2011) [1] | UK | - Confirmed (H1N1)2009 patients: n=186 - ILI cases (non-(H1N1)2009): n=83 | - Confirmed (H1N1)2009 patients: 0.96 (min-max: 0.15-1) - ILI cases (non-(H1N1)2009):   0.97 (min-max: 0.15-1) |
| Hollmann, M  (2013) [3] | Spain | Confirmed (H1N1)2009 patients (aged 8 years old and over):   - Inpatients: n=432 - Outpatients: n=563 | - Inpatients: 0.81 (95%CI, 0.78-0.84) - Outpatients: 0.93 (95%CI, 0.91-0.96) |
| Liu Y (2010) [4] | Western China | General population (aged 15 years old and above, n=2026) | - 0.8 |

[1] van Hoek AJ, Underwood A, Jit M MIller E, Edmunds WJ. The impact of pandemic influenza H1N1 on health-related quality of life: a prospective population based study. PLoS One 2011; 6(3): e17030. doi: 10.1371/journal.pone.0017030.

[2] Griffin AD, Perry AS, Fleming DM. Cost-effectiveness analysis of inhaled zanamivir in the treatment of influenza A and B in high-risk patients. Pharmacoeconomics 2001;19(3):293-301.

[3] Hollmann M, Garin O, Galante M, Ferrer M, Dominguez A, Alonso J. Impact of influenza on health-related quality of life among confirmed (H1N1)2009 patients. PLoS One 2013; 8(3): e60477. doi: 10.1371/journal.pone.0060477.

[4] Liu Y, Gao J: Study on the health-related quality of life of rural residents in western China. [in Chinese] Chinese Medical Ethics 2010, 23(4):100-101,105.
