# Supplementary figures and images for: The impact of influenza on the health related quality of life in China: an EQ-5D survey
Source: BMC Infect Dis. 2017 Oct 16;17:686. doi: 10.1186/s12879-017-2801-2 (PMC5644056; doi:10.1186/s12879-017-2801-2)

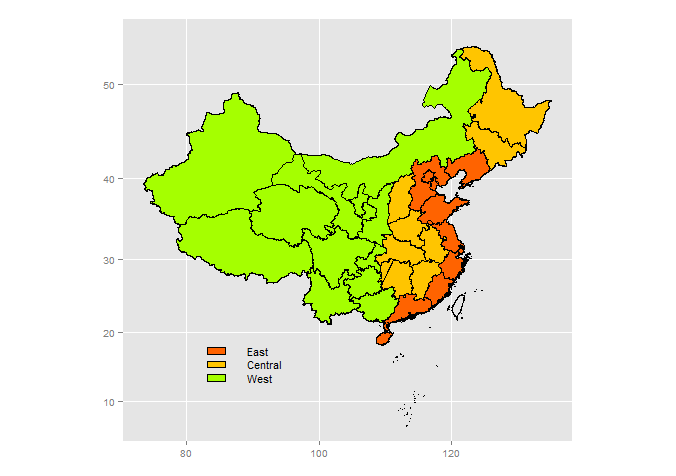

Supplement: Supplementary file 2 — Map of geographic regions in mainland China. (TIFF 1033 kb) [file 12879_2017_2801_MOESM2_ESM.tif]
