# Supplementary material for: The impact of influenza on the health related quality of life in China: an EQ-5D survey
Source: BMC Infect Dis. 2017 Oct 16;17:686. doi: 10.1186/s12879-017-2801-2 (PMC5644056; doi:10.1186/s12879-017-2801-2)
Supplement: Supplementary file 3 — Definition of hospital levels. (DOCX 19 kb) [file 12879_2017_2801_MOESM3_ESM.docx]

**Additional file 3: Definition of hospital levels**

According to the Basic Standard of Medical Institutes (http://www.nhfpc.gov.cn/mohzcfgs/pgz/200804/18713.shtml) issued by National Health and Family Planning Commission of China, the hospitals are broken down into three levels in China, defined as follows:

**1. Level 3**

(1) Beds: 500 beds and above;

(2) Departments: clinical departments at least include emergency department, internal medicine department, department of surgery, obstetrics and gynecology department, and department of pediatrics, etc.; medical technical departments at least include pharmacy department, clinical laboratory, radiology department, and operating room, etc.

(3) Personnel: at least 1.03 health technicians and 0.4 nurses per bed; the director of each department should at least have the professional title of associate chief physicians; at least 2 clinical nutritionists; engineering technicians account for at least 1% of all health technicians;

(4) Buildings: floor area is at 60 square meters per bed; usable area is at least 6 square meters per bed; etc.

(5) Equipment: basic equipment (e.g., ventilator, electrocardiograph, and anesthesia machine); equipment for each bed (same with those mentioned in Level 1 hospitals); other specific equipment for the conducted diagnosis and treatment.

**2. Level 2**

(1) Beds: 100-499 beds and above;

(2) Departments: clinical departments at least include emergency department, internal medicine department, department of surgery, obstetrics and gynecology department, and department of pediatrics, etc.; medical technical departments at least include pharmacy department, clinical laboratory, radiology department, and operating room, etc.

(3) Personnel: at least 0.88 health technicians and 0.4 nurses per bed; at least 3 doctors have the professional title of associate chief physicians; at least 1 doctor has the professional title of attending physician in each professional department;

(4) Buildings: floor area is at 45 square meters per bed; usable area is at least 5 square meters per bed; etc.

(5) Equipment: basic equipment (e.g., ventilator, electrocardiograph, and anesthesia machine); equipment for each bed (add 1 bed lamp besides those mentioned in Level 1 hospitals); other specific equipment for the conducted diagnosis and treatment.

**3. Level 1**

(1) Beds: 20-99 beds and above;

(2) Departments: clinical departments at least include emergency department, internal medicine department, obstetrics and gynecology department, and department of preventive medicine; medical technical departments at least include pharmacy department, clinical laboratory, radiology department, and disinfection supply division.

(3) Personnel: at least 0.7 health technicians per bed; at least 3 doctors, 5 doctors and technicians in such fields like pharmacy, laboratory, and radiology; at least 1 doctor has the professional title of attending physician in each professional department ;

(4) Buildings: floor area is at 45 square meters per bed;

(5) Equipment: basic equipment (e.g., ventilator, electrocardiograph, and anesthesia machine); equipment for each bed (e.g., 1 bed, 1.2 mattress, 1.2 quilts); other specific equipment for the conducted diagnosis and treatment.
