# Supplementary material for: The impact of influenza on the health related quality of life in China: an EQ-5D survey
Source: BMC Infect Dis. 2017 Oct 16;17:686. doi: 10.1186/s12879-017-2801-2 (PMC5644056; doi:10.1186/s12879-017-2801-2)
Supplement: Supplementary file 4 — Characteristics of included patients and other lab-confirmed influenza patients from the National ILI Surveillance Network in the survey. (DOCX 25 kb) [file 12879_2017_2801_MOESM4_ESM.docx]

**Additional file 4: Table S1. Characteristics of included patients and other lab-confirmed influenza patients from the National ILI Surveillance Network in the survey**

| Characteristic | Included patients in the survey  (n=778) | Other lab-confirmed influenza patients from surveillance networks  (n=39190) | Test for difference in groups | *p*-value |
| --- | --- | --- | --- | --- |
| Median age, years (inter-quartile range) | 7(3-15) | 9(4-27) | Rank-sum test | <0.001 |
| Age group, years (%) |  |  | Chi-square test | <0.001 |
| <5 | 263 (33.8) | 11821 (30.2) |  |  |
| 5-15 | 326 (41.9%) | 12101 (30.9) |  |  |
| 16-59 | 170 (21.9%) | 13636 (34.8) |  |  |
| ≥60 | 19 (2.4) | 1632 (4.2) |  |  |
| Male (%) | 425 (54.6) | 21518 (54.9) | Chi-square test | 0.905 |
| Region (%) |  |  | Chi-square test | 0.846 |
| East China | 384 (49.4) | 18936 (48.3) |  |  |
| Central China | 200 (25.7) | 10317 (26.3) |  |  |
| West China | 194 (24.9) | 9937 (25.4) |  |  |
| Hospital (%) |  |  | Chi-square test | <0.001 |
| Level 1 and lower | 131 (16.8) | 2682 (6.8) |  |  |
| Level 2 | 177 (22.8) | 9964 (25.4) |  |  |
| Level 3 | 470 (60.4) | 26544 (67.7) |  |  |
