# Supplementary material for: The impact of influenza on the health related quality of life in China: an EQ-5D survey
Source: BMC Infect Dis. 2017 Oct 16;17:686. doi: 10.1186/s12879-017-2801-2 (PMC5644056; doi:10.1186/s12879-017-2801-2)
Supplement: Supplementary file 5 — Comparison of the health-related quality of life for influenza patients aged 16 years and above between the self-report and proxy-report groups. (DOCX 22 kb) [file 12879_2017_2801_MOESM5_ESM.docx]

**Additional file 5: Table S2. Comparison of the health-related quality of life for influenza patients aged 16 years and above between the self-report and proxy-report groups**

|  |  | Outpatient | | |  | Inpatient | | |
| --- | --- | --- | --- | --- | --- | --- | --- | --- |
|  |  | Proxy-report (n=36) | Self-report (n=125) | Multiple linear regression  (95%CI) ^a^ |  | Proxy-report (n=7) | self-report (n=21) | Multiple linear regression  (95%CI) ^a^ |
| VAS | Mean (SD) | 69.58  (17.62) | 64.94  (19.43) | -6.09  (-14.45, 1.34) |  | 62.86 (24.98) | 64.19 (22.72) | -8.04  (-26.26, 10.80) |
|  | Median(IQR) | 70.00  (60.00, 80.00) | 70.00  (50.00, 80.00) |  |  | 50.00 (50.00,80.00) | 60.00 (50.00,80.00) |  |
| Health utility | Mean (SD) | 0.5868  (0.2261) | 0.5934  (0.2132) | -0.0001  (-0.0985, 0.0978) |  | 0.3913  (0.2805) | 0.5097  (0.2768) | -0.0120  (-0.1985, 0.1659) |
|  | Median(IQR) | 0.6825 (0.4308,0.7305) | 0.6430 (0.4510,0.7290) |  |  | 0.3650  (0.1395,0.6430) | 0.5630  (0.3650,0.7290) |  |
| QALD loss | Mean (SD) | 1.60  (1.84) | 1.83  (2.34) | 0.25  (-0.75, 1.20) |  | 9.22 (8.21) | 4.53 (6.03) | -2.43  (-7.06, 4.77) |
|  | Median(IQR) | 0.89  (0.48,2.02) | 1.09  (0.41,2.48) |  |  | 6.51 (4.35,12.07) | 2.08 (1.31,4.45) |  |

^a^ Multiple linear regression analysis was used to control for other covariates (including hospital level, region, age, area, gender, influenza virus type, and underlying disease). The results showed the absolute increase or decrease of the VAS, health utility and/or QALD loss compared to the proxy-report group. And we obtained the bias-corrected and accelerated (BCa) bootstrap percentile confidence interval using the R function “boot.ci”.
