# Supplementary material for: The impact of influenza on the health related quality of life in China: an EQ-5D survey
Source: BMC Infect Dis. 2017 Oct 16;17:686. doi: 10.1186/s12879-017-2801-2 (PMC5644056; doi:10.1186/s12879-017-2801-2)
Supplement: Supplementary file 6 — Health-related quality of life of influenza patients stratified by time delay between survey and the influenza episode. (DOCX 32 kb) [file 12879_2017_2801_MOESM6_ESM.docx]

**Additional file 6: Table S3. Health-related quality of life of influenza patients stratified by time delay between survey and the influenza episode**

|  | N | VAS | |  | Health utility | |  | QALD loss^a^ | |
| --- | --- | --- | --- | --- | --- | --- | --- | --- | --- |
|  |  | Mean  (SD) | Median  (IQR) |  | Mean  (SD) | Median  (IQR) |  | Mean  (SD) | Median  (IQR) |
| Influenza outpatients |  |  |  |  |  |  |  |  |  |
| Time delay between survey and the actual influenza episode (days) | | | | | | | | | |
| Unweighted |  |  |  |  |  |  |  |  |  |
| 0-20 | 167 | 68.13  (17.30) | 70.00 (60.00,80.00) |  | 0.6251 (0.1969) | 0.6430  (0.5690,0.7350) |  | 1.59 (1.82) | 1.09 (0.50,2.01) |
| 21-46 | 154 | 69.08 (19.30) | 70.00 (60.00,80.00) |  | 0.6020  (0.2109) | 0.6430  (0.5645,0.7290) |  | 1.63  (1. 80) | 1.12 (0.40,2.08) |
| 47-103 | 122 | 70.56 (19.17) | 70.00 (60.00,80.00) |  | 0.6178 (0.2037) | 0.6430  (0.5645,0.7350) |  | 1.73 (2.19) | 1.09 (0.46,2.04) |
| ≥103 | 86 | 67.93 (21.82) | 70.00 (60.00,80.00) |  | 0.6095 (0.1860) | 0.6430  (0.5488,0.7290) |  | 1.52 (1.38) | 1.24 (0.51,2.07) |
| Weighted^b^ |  |  |  |  |  |  |  |  |  |
| 0-20 | 167 | 65.58 (35.12) | 60.83 (37.90,81.86) |  | 0.5965 (0.3383) | 0.5725 (0.3287,0.7039) |  | 1.60 (2.42) | 0.87 (0.34,1.81) |
| 21-46 | 154 | 65.04 (30.98) | 62.31 (43.45,80.75) |  | 0.5699 (0.3144) | 0.5588 (0.3467,0.7474) |  | 1.63 (2.39) | 1.04 (0.35,2.02) |
| 47-103 | 122 | 69.23 (39.33) | 60.83 (37.90,81.86) |  | 0.6167 (0.4018) | 0.5588  (0.3172,0.7996) |  | 1.84 (3.29) | 0.86 (0.41,1.77) |
| ≥103 | 86 | 77.48 (39.79) | 76.94 (52.14,100.89) |  | 0.6904 (0.3576) | 0.6165 (0.4296,0.9189) |  | 1.73 (1.89) | 1.34 (0.51,2.21) |
| Influenza inpatients |  |  |  |  |  |  |  |  |  |
| Time delay between survey and the actual influenza episode (days) | | | | | | | | | |
| Unweighted |  |  |  |  |  |  |  |  |  |
| 0-20 | 35 | 66.14 (20.15) | 70.00 (60.00,80.00) |  | 0.5897 (0.1892) | 0.6430 (0.4280,0.7290) |  | 2.77 (2.56) | 2.33 (1.28,3.2) |
| 21-46 | 40 | 59.00 (19.29) | 60.00 (50.00,70.00) |  | 0.5674 (0.1865) | 0.6060 (0.5415,0.6430) |  | 3.44 (2.79) | 2.33 (1.52,4.81) |
| 47-103 | 74 | 66.43 (19.27) | 70.00 (50.00,80.00) |  | 0.5919 (0.2273) | 0.6430 (0.5690,0.7350) |  | 3.71 (5.85) | 1.67 (0.88,3.74) |
| ≥103 | 100 | 68.29 (21.89) | 70.00 (50.00,86.25) |  | 0.5855 (0.2380) | 0.6430 (0.5020,0.7350) |  | 3.65 (3.80) | 2.60 (0.88,4.96) |
| Weighted^b^ |  |  |  |  |  |  |  |  |  |
| 0-20 | 35 | 65.42 (27.05) | 69.52 (53.33,78.22) |  | 0.5765 (0.2359) | 0.5643 (0.3774,0.7474) |  | 2.63 (2.33) | 2.15 (1.10,3.51) |
| 21-46 | 40 | 59.28 (36.26) | 54.52 (34.89,69.52) |  | 0.5687 (0.3226) | 0.5497 (0.4039,0.6379) |  | 3.30 (2.75) | 2.81 (1.30,4.38) |
| 47-103 | 74 | 62.19 (24.84) | 60.83 (44.46,72.43) |  | 0.5360 (0.2416) | 0.5588 (0.4242,0.6388) |  | 4.21 (8.53) | 1.68 (0.76,3.22) |
| ≥103 | 100 | 66.91 (29.44) | 66.34 (43.45,84.18) |  | 0.5724 (0.3040) | 0.5588 (0.3841,0.7474) |  | 3.68 (4.18) | 2.44 (0.84,5.61) |

^a^ QALD: quality-adjusted life days.

^b^ Weighted: Previous analysis showed that population structure (by age group, region and levels of hospitals) of included patients was significantly different from that of other influenza patients from the National ILI Surveillance Network [S4 Table]. Hence we calculated the weighted HRQoL of included patients using the population structure of the National ILI Surveillance Network as a reference.
